# Ferritin in cancer therapy: A pleiotropic tumoraffin nanocage‐based transport

**DOI:** 10.1002/cam4.5778

**Published:** 2023-03-31

**Authors:** Guodong Deng, Yang Li, Ning Liang, Pingping Hu, Yan Zhang, Lili Qiao, Yingying Zhang, Jian Xie, Hui Luo, Fei Wang, Fangjie Chen, Fengjun Liu, Deguo Xu, Jiandong Zhang

**Affiliations:** ^1^ Department of Oncology The First Affiliated Hospital of Shandong First Medical University and Shandong Province Qianfoshan Hospital, Shandong Lung Cancer Institute Jinan China; ^2^ Department of Oncology Shandong First Medical University Jinan China; ^3^ Department of Oncology The Affiliated Cancer Hospital Of Zhengzhou University Zhengzhou China; ^4^ Department of Oncology Zaozhuang Shizhong District People's Hospital Zaozhuang China

**Keywords:** cancer, cancer treatment, drug delivery, ferritin

## Abstract

**Background:**

Ferritin, a ubiquitously distributed iron storage protein, can specifically target tumor cells through transferrin receptor 1. Due to its rearrangeable nanocage structure, ferritin can be loaded with anticancer drugs. Combined with amino acid modifications on the outer‐ and/or inner‐spaces of the nanocage, ferritins can be further coupled with antigens, antibodies, and nucleotide sequences. Since ferritin is naturally presented in the human body, when used in vivo, ferritin exhibits good biocompatibility, and no immunogenic response occurs. These makes ferritin an ideal nanocarrier which shows broad application prospects in cancer therapy.

**Methods:**

In this study, to find articles, a search was made in PubMed with the keywords ferritin, drug delivery, drug delivery, and cancer treatment.

**Results:**

According to the investigation, some studies suggest that ferritin can be loaded with drugs and targeted for delivery to tumor tissue. Therefore, ferritin nanocarriers loaded with drugs can be used in chemotherapy, photodynamic therapy (PDT), photothermal therapy (PTT) and immunotherapy. Importantly, the specific targeting of ferritin nanocarriers to tumor cells increases the effectiveness of related therapies and reduces side effects.

**Conclusions:**

We conclude in this paper that the superior properties of ferritin nanocarriers as an emerging drug delivery system make them a promising cancer treatment strategy. In the future, it is worth conducting clinical trials to further investigate the safety and efficacy of ferritin nanocarriers in patients.

## Graphical abstract

Ferritin nanocarriers have a good prospect in the treatment of cancer. Based on the specific targeting of ferritin to tumor cells, ferritin nanocarriers can deliver drugs to mouse tumor tissues by loading therapeutic drugs or modifying them on the surface of ferritin. This allows for precise treatment of tumors with fewer side effects. In conclusion, ferritin nanocarriers can provide a promising therapeutic strategy for the treatment of tumors.

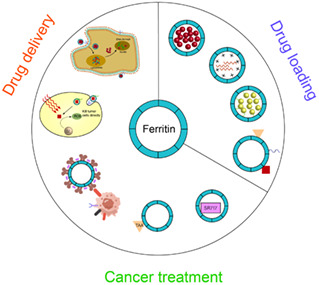

## 1 INTRODUCTION

Cancer is currently a major unsolved health problem, and the treatment of cancer mainly includes traditional postoperative radiotherapy and chemotherapy as well as cutting‐edge immunotherapy.^1^, ^2^, ^3^ However, as research has progressed, the limitations of the treatment have been exposed. For example, both chemotherapeutic drugs and therapeutic antigens have inherent problems such as short half‐phase, insufficient targeting ability and potential systemic toxicity.^4^, ^5^ Excitingly, nanocarrier researches offer promising strategies to address these issues and improve the anti‐tumor efficacy.

Human ferritin, whose gene is located on chromosomes 11q and 19q, consists of two distinct subunits. The light chain (L‐ferritin) and heavy chain (H‐ferritin) are 19 and 21 kDa in size, respectively. The two subunits share approximately 55% sequence homology and share a similar three‐dimensional structure, including four parallel and antiparallel helices (a–d) and a fifth shorter e‐helix.^6^, ^7^, ^8^ A typical ferritin consists of 24 subunits that aggregate and self‐assemble to form an almost spherical hollow nanocages. The inner and outer cavities of this nanocage exhibit diameters of 8 nm and 12 nm, respectively, and can bind about 4500 iron atoms.^7^, ^9^ Ferritin is ubiquitous distributed in different organs, by adjusting the labile iron pool, it plays an important role in regulating iron metabolism and iron‐related biological processes.^6^, ^10^

Intracellular ferritin can protect cells from Fenton reaction by oxidizing toxic ferrous ions to ferric ions and storing them.^7^ Because Fenton reaction produces reactive oxygen species, lipid peroxidation can induce ferroptosis, so ferritin can also inhibit the occurrence of ferroptosis.^11^, ^12^, ^13^ In addition, ferritin can also regulate t he energy balance and thermogenesis of the organism.^14^ In the clinic, the concentration of serum ferritin is the most used indicator to determine iron deficiency in vivo. Importantly, serum ferritin can also be used as an assessment marker for cancer diagnosis and prognosis.^6^ Therefore, nanocarriers with tumor‐targeting properties show great potential in tumor therapy. Ferritin, due to its unique structure, properties, and high biocompatibility, plays an important role in the treatment of cancer.

**FIGURE 1:**
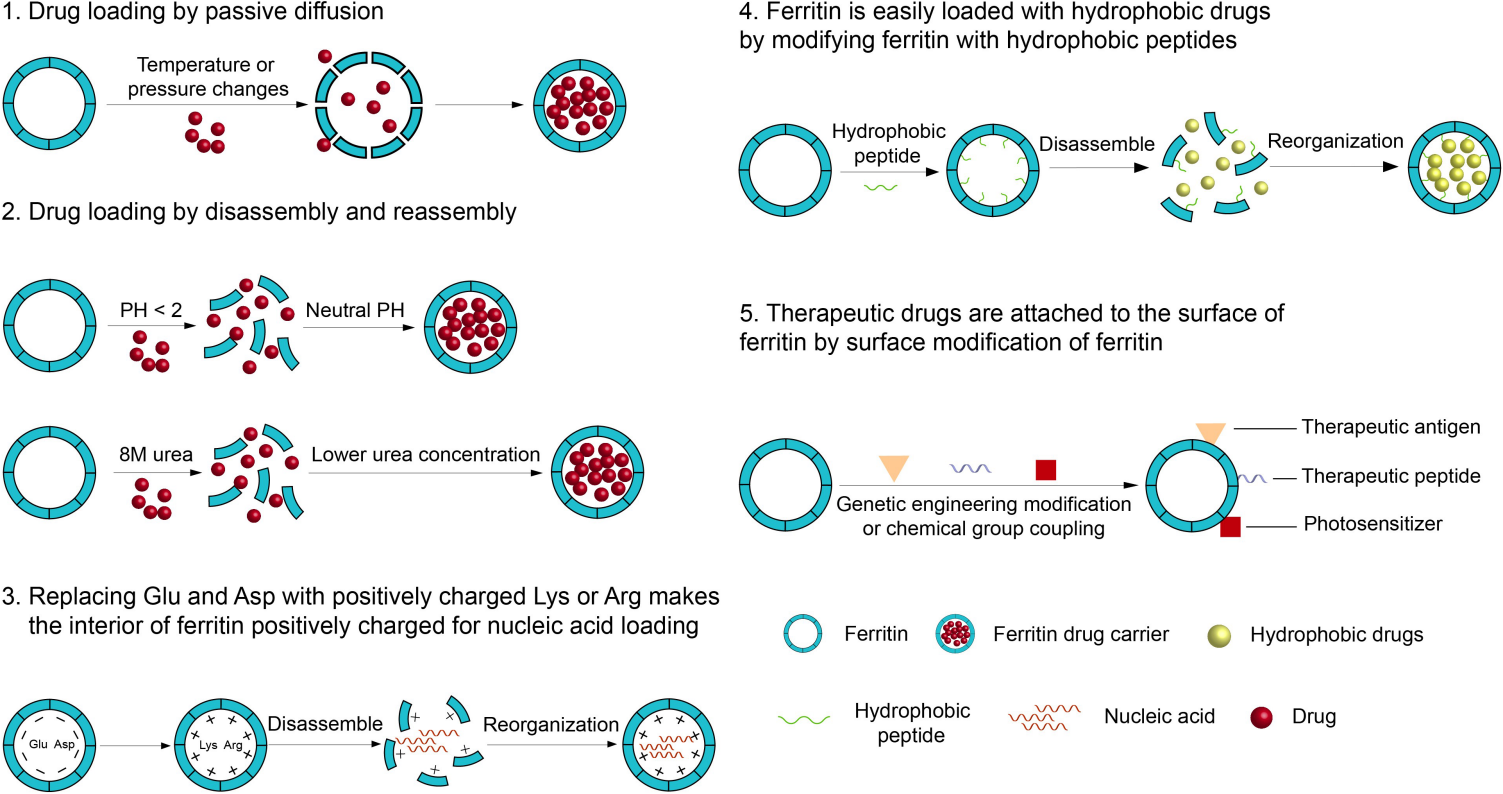
Drug loading methods of ferritin.

Thorough disassembly/reassembly of ferritin, drugs can be loaded into the nanocage cavity, while different regulation manners are accessible for this process. Ferritin subunits were separated in pH < 1.96 solution and in 8 M urea, after which the ferritin nanocages can be reassembled and loaded with drugs by increasing the pH and adjusting the urea gradient^15^, ^16^ (Figure 1). In addition, drugs can also be loaded by passive diffusion (Figure 1). The ferritin nanocages contain 6 hydrophobic quadruple (C4) channels, each formed by a helix of 4 monomers, and 8 narrow hydrophilic triplet (C3) channels.^17^ The channel structure and schematic diagram of ferritin nanocarriers are described in detail in the review by Marina et al.^6^ By increasing the temperature, the channels in the ferritin structure can be opened or expanded, and small molecule drugs could pass through the channels into the ferritin cage.^18^, ^19^, ^20^ Thus, drugs could be efficiently loaded into ferritin nanocages. Doxorubicin (DOX), epirubicin, cisplatin and oxaliplatin have been loaded into ferritin cages based on heat‐induced passive diffusion.^20^ Studies have shown that ferritin nanocages can also be effectively loaded with photosensitizers, therapeutic nucleic acids, fluorescent molecules or contrast agents.^16^, ^21^, ^22^ In addition, ferritin nanocages can also be easily modified by genetic engineering or chemical reactions to introduce therapeutic substances and additional functions.^23^ For example, PD‐L1‐binding peptides and SIRP variants are modified on the surface of ferritin for immunotherapy.^24^, ^25^

In addition to ferritin nanocarriers, various nanocarriers have been developed for loading different kinds of drugs or contrast agents (e.g., liposomes, polymer nanoparticles, proteins, carbon nanotubes (CNTs), and gold nanoparticles).^26^, ^27^ These nanocarriers can be passively accumulated in tumor tissues due to increased permeability and retention effect of tumor tissues.^28^ This is hailed as a huge advantage for all types of nanocarriers.^29^ In addition to passive targeting, it is exciting that ferritin nanocarriers can accumulate in tumor areas by both passive and active targeting. Studies have shown that human heavy chain ferritin (HFn) can specifically target the transferrin receptor 1 (TfR1) overexpressed by tumor cells.^30^, ^31^ And human HFn can enter tumor cells through receptor‐mediated internalization after interacting with TfR1.^15^, ^32^ This makes ferritin nanocarriers superior to other types of protein‐based nanocarriers (such as serum albumin). Therefore, ferritin nanocarriers loaded with drugs can be used for targeted therapy of cancer cells. Moreover, ferritin can tolerate at 80°C for 10 minutes while maintaining its structural integrity and stability.^33^ So, we can purify ferritin by heating, which simplifies the preparation of ferritin compared to other nanocarriers. Most importantly, ferritin occurs naturally in the human body. Therefore, it will show excellent biocompatibility when used in vivo, which makes the ferritin carrier hardly cause side effects.^15^ The good biocompatibility of ferritin nanocarriers also makes it better than inorganic nanocarriers (e.g., Au‐based, SiO_2_‐based, MoS2‐based, phosphorus‐based nanoplatforms).

Therefore, the excellent structure and properties of ferritin make it great potential in tumor treatment (Table 1). Ferritin nanocarriers can deliver chemotherapeutic drugs and reduce chemotherapy side effects by their superior loading capacity and tumor targeting. In recent years, advanced studies have shown that bioengineered ferritin could act as an important carrier for activating immunity, playing an important role in immunotherapy, and can enables a combination of multiple treatments strategies. In this review, we mainly introduce ferritin nanoparticles for cancer chemotherapy, immunotherapy, photodynamic, photothermal therapy and the combined therapies.

**TABLE 1:** Ferritin for cancer treatment.

Drug carrier	Drug	Drug injection equivalent	Treatment method	Cancer type	Treatment effect	References
Human HFn	DOX	DOX: 10 mg/kg × 6 times	Chemotherapy	Hepatocellular carcinoma	Has completely inhibited tumor growth	20
Human HFn	DOX	—	Chemotherapy	Gastric cancer	Has increased TGI rate (91.1%) compared to free DOX (41.6%)	34
Human HFn	PTX	PTX: 4 mg/kg, 10 times/2 days	Chemotherapy	Breast cancer	Has significantly reduced tumor volume gain (0.8 cm^3^) compared to the PBS group (2.26 cm^3^)	35
Horse Spleen Apoferritin	CUR and QUE	—	Chemotherapy	Breast cancer	In the MCF7 cell animal model, co‐administration significantly reduced EC50 (11 μM) compared to CUR or QUE alone (24 and 47.5 μM)	36
Human HFn	DOX	DOX: 20 mg/kg × 1 times	Chemotherapy	Colon cancer	The 40‐day survival rate of the HT‐29 tumor model was 83.3%	15
Human HFn	DOX	DOX: 1 mg/kg, 3 times/3 days	Chemotherapy	Glioma	The median survival time of mice was increased to 30 days with less side effects than free DOX	37
Human HFn	PTX	PTX: 1 mg/kg × 5 times	Chemotherapy	Glioma	The median survival time of mice increased to 30 days significantly longer than free PTX (14 days) treated mice	38
Human HFn (Hydrophobic polypeptide modified)	CPT and EPI	CPT and EPI: Glioma: 35 mg/kg × 3 times; liver cancer and breast cancer: 35 mg/kg × 2 times	Chemotherapy	Glioma, liver cancer and breast cancer	Completely inhibited the growth of U87MG, HepG2 and MCF7‐MDR tumor models	39
Human HFn (GE11 peptides, CTSE cleavage sequence and (DE)_3_ modified)	GEM and DOX	GEM: 1.5 mg/kg × 13 times; DOX: 0.5 mg/kg × 13 times	Chemotherapy	Pancreatic cancer	No toxicity to normal cells	40
Human HFn (RGD peptide modified)	DOX	DOX: 5 mg/kg × 5 times	Chemotherapy	Glioblastoma	Has increased TGI (89.6%) compared to free DOX	41
Human HFn (Penetrating peptide tLyP‐1 modified)	PTX	PTX: 1 mg/kg for 20 days	Chemotherapy	Breast cancer and liver cancer	Has enhanced the tumor penetration ability of the nanocarriers	42
Apoferritin (GKRK peptide modified)	VCR	VCR: 1 mg/kg, 4 times/2 days	Chemotherapy	Glioma	Has enhanced the ability of ferritin to cross the BBB	43
Pyrococcus furiosus ferritin (SP94 modified)	DOX	DOX: 25 mg/kg × 2 times	Chemotherapy	Hepatocellular carcinoma	Has prompted the survival time of mice (90 days), compared with DOX alone (<36 days), and also significantly inhibited tumor lung metastasis	44
Human HFn (Integrin α2β1 targeting peptide modified)	DOX	DOX: 1 mg/kg × 2 times	Chemotherapy	Glioma	Has enhanced the drug loading capacity of ferritin and its ability to cross the BBB	45
Human HFn (PAS peptide modified)	DOX	DOX: 5 mg/kg	Chemotherapy	Pancreatic cancer	Has increases the half‐life of ferritin nanocarriers	46
Human HFn (PASE peptide modified)	Genz‐644,282	Genz‐644,282: 1.9 mg/kg × 6 times	Chemotherapy	Pancreatic cancer、breast cancer and liver cancer	The TGI and ORR of PaCa44, MDA‐MB‐231 and HepG2 tumor‐bearing mice were all 100%	47
Human HFn (PAS peptide and RGDK peptide modified)	DOX	DOX: 3.0 mg/kg	Chemotherapy	Breast cancer	Has increased the amount of drug accumulated in the tumor area	48
Human HFn (Poly ethylene glycol modified)	Acriflavine and Cisplatin	Acriflavine and Cisplatin: 2.0 mg/kg	Chemotherapy	Lung cancer	Restored chemosensitivity of cisplatin and increased median survival time in mice	49
Human HFn (Collagenase nanocapsules modified)	DOX	DOX: 5 mg/kg × 7 times	Chemotherapy	Breast cancer	Tumor inhibition rate reached 85%	50
Short ferritin (CLT and microplasmin (μP) proteins modified)	DOX	DOX: 1 mg/kg × 5 times	Chemotherapy	Melanoma and breast cancer	Improved drug distribution in tumors. The therapeutic effect was better than DOX alone in both B16F10 and MDA‐MB‐231 tumor models	51
Apoferritin	Gefitinib	—	Target therapy	Breast cancer	Has enhanced resistance to SKBR3 breast cancer cells (GI50 = 0.52 × 10^−6^ M) compared to gefitinib alone (GI50 = 1.66 × 10^−6^ M)	52
Ferritin	Chlorin e6 and MnO_2_	—	PDT	Breast cancer	Improved hypoxic tumor microenvironment and enhanced PDT efficacy compared to Ce6 alone	22
Rat heavy chain ferritin (CGKRK peptide modified)	556‐Ph	556‐Ph: 5 mg/kg	PTT and PDT	Breast cancer	PDT + PTT treatment resulted in complete tumor eradication and no recurrence within 16 days	53
Apoferritin	RSV and IR780	RSV: 5 mg/kg × 2 times; IR780: 2 mg/mL	PTT and Chemotherapy	Ovarian cancer	Two months after initial treatment, mice survived 100%	54
Horse spleen apoferritin (FA modified)	IR1061 and PTX	IR‐AFN@PTX‐FA: 5 or 10 mg/kg	PTT and Chemotherapy	Breast cancer	Laser had deeper tissue penetration and could significantly inhibit tumor growth	55
Human HFn	ATO and ICG	ATO: 5 mg/kg × 5 times; ICG: 0.8 mg/kg × 5 times	PTT and Chemotherapy	Cervical cancer, stomach cancer and breast cancer	Has completely inhibited tumor growth in HeLa xenograft mice and reduced tumor volume to 150 cm^3^ in MCF‐7 and MGC‐803 mouse models	56
Human HFn	RFP	HFn‐RFP: 10 μ l	Immunotherapy	All types	Has generated tumor antigen‐specific cytotoxic CD8+ T cells	57
Human HFn (M2‐macrophage targeting peptide modified)	CpG oligonucleotide	CpG: 11.08 μg × 4 times	Immunotherapy	Breast cancer	Has polarized M2‐type macrophages to M1‐type and significantly inhibited tumor growth	58
Human HFn (Tumor targeting peptide RGE modified)	STING agonist SR‐717	SR‐717: 5 mg/kg × 5 times	Immunotherapy	Glioma	Has reduced tumor volume by 55.3% compared to PBS control	59
Human HFn (SIRPγ variant modified)	SIRPγ variant and CpG	FSγV: 10 mg/kg × 3 times	Immunotherapy	Melanoma	Has increased the ratio of dendritic cells and CD8+ T cells in the TME and suppressed tumor volume by 82.5%	60
Human HFn (Extracellular domain gene of PD‐1 modified)	Extracellular domain gene of PD‐1	PdNC: 23.7 mg/kg	Immunotherapy	Colon cancer	Has induced DC‐mediated T cell activation and suppressed tumor volume by 75%	61
Rat heavy chain ferritin (scFv sequence modified)	ZnF16Pc	—	Immunotherapy and PDT	Breast cancer	Has broken down the ECM to enhance the diffusion of ferritin nanoparticles in the tumor	62
Human HFn (Photosensitizer Ce6 modified)	CpG	—	Immunotherapy and PDT	Breast and lung cancer	In a 4 T1 cell mouse model, this reduced the number of lung metastases (14 ± 12) compared to HFn alone(101 ± 46)	21
Human ferritin (SIRPα variant modified)	SIRPα variant and DOX	DOX: 1 mg/kg	Immunotherapy and Chemotherapy	Melanoma and colon cancer	Completely eradicated CL25 and B16 cell‐type tumors in mice	24
Human HFn (SIRPα variant modified)	SIRPα variant and MPD‐1	FS: 10 mg/kg × 3 times; MPD‐1: 5 mg/kg × 4 times	Immunotherapy and Chemotherapy	Colon cancer	Completely eradicated tumors in 8 of 9 mice and elicited tumor‐specific memory	63
Human HFn (PD‐L1 binding peptide 1 modified)	DOX and PD‐L1 binding peptide 1	PpNF(DOX): 10 mg/kg × 8times	Immunotherapy and Chemotherapy	Colon cancer	Significantly improved tumor suppression compared to DOX and anti‐PD‐L1 antibody alone	25
Apoferritin (Anti‐FAP single chain antibody modified)	Photosensitizer ZnF16Pc	ZnF16Pc: 1.5 mg/kg	Immunotherapy and PDT	Breast cancer	Disrupted the ECM layer outside the tumor and significantly improved T cell infiltration	64
Apoferritin (Anti‐FAP single chain antibody modified)	ZnF16Pc and anti‐PD‐1 antibody	ZnF16Pc: 0.5 mg/kg; anti‐PD‐1: 10 mg/kg × 3 times	Immunotherapy and PDT	Breast cancer	Increased mean animal survival time (42.4 days) compared to anti‐PD‐1 antibody alone(29.4 days)	65

Abbreviations: ATO, arsenic trioxide; CPT, camptothecin; GKRK, glioma biomarker heparan sulfate proteoglycan targeting peptide; CGKRK, targeting peptide for high expression of heparin sulfate on the surface of tumor cells; CTSE, non‐secretory aspartic protease highly expressed in tumor cells; CLT, fibrin clot‐targeting peptide; CUR, curcumin; DOX, doxorubicin; (DE)_3_, carboxyl Rich Peptides; EPI, epirubicin; ECM, extracellular matrix; EC50, median effect concentration; EGFR, epidermal growth factor receptor; FSγV, HFn modified by SIRPγ variant; FS, HFn modified by SIRPα variant; FA, folic acid; GEM, gemcitabine; Genz‐644,282, wide‐spectrum topoisomerase I inhibitor; HFn, heavy chain ferritin; IR‐AFN@PTX‐FA, FA‐modified ferritin loaded with IR1061 and PTX; ICG, indocyanine green; MPD‐1, caspase‐cleavable peptide‐doxorubicin conjugate; ORR, objective response rate; PTX, paclitaxel; PBS, phosphate buffered saline; PAS, polypeptide sequences rich in proline (P), serine (S) and alanine (a) residues; PASE, insertion of two glutamic acid residues in the PAS sequence (E); PTT, photothermal therapy; PTD, photodynamic therapy; PdNC, PD‐1‐decorated nanocages; PpNF, HFn modified by PD‐L1 binding peptide 1; PpNF(DOX), PpNF loaded with DOX; QUE, quercetin; RFP, red fluorescence protein; RGE, glioma targeting peptide; RSV, resveratrol; RGD, three amino acid sequences targeting integrin Rvβ3; RGDK, integrin αvβ5 targeting peptide; scFv, single‐chain variable fragment of fibroblast activation protein; STING, stimulator of interferon genes; SIRPγ, signal regulatory proteins γ; SIRPα, signal regulatory proteins α; TGI, tumor growth inhibition rate; tLyP‐1, glioma targeting peptide; TME, tumor microenvironment; VCR, vincristine.

## 2 OVERVIEW OF DRUG LOADING METHODS OF FERRITIN

As mentioned before, the drug loading methods of ferritin nanocarriers mainly include passive diffusion and disassembly and recombination methods.^15^, ^16^, ^52^ However, the above methods have certain limitations. Methods based on passive diffusion need to consider the diameter of the channel and drug molecules, and the drug can enter the nanocages smoothly, so its drug‐carrying capacity is limited. The pH‐based disassembly /recombination method would destroy the structure of ferritin in a very acidic environment, and could not restore the original hollow spherical structure, but formed a hollow spherical structure with two holes, which would also affect the recovery rate of ferritin.^16^, ^66^ Recently, a method based on thermal induction was developed. Enlarging the channels in the ferritin cage by increasing the temperature could enable chemotherapy drugs to be loaded into the ferritin cage by passive diffusion. This method significantly improved drug loading and ferritin recovery.^19^, ^20^ In passive diffusion, the diffusion of drugs in ferritin cages depends on the respective charge and polarity.^67^ However, the presence of most hydrophilic residues on the inner surface of ferritin hinders the loading of hydrophobic drugs.^20^ To solve this problem, Wang et al.^39^ reconstructed the inner surface of ferritin nanocages by genetically fusing hydrophobic polypeptides to the C‐terminus of human heavy chain ferritin (HFn) subunits. Similarly, nucleic acids exhibiting a negative charge are also difficult to load into ferritin due to the negative charge of the ferritin lumen.^68^ Zhang et al.^21^ constructed an internally positively charged ferritin (HFn(+)) by replacing the negatively charged glutamic acid and aspartic acid in the ferritin lumen with positively charged lysine or arginine. Afterwards, the nucleic acid loading was significantly increased by disassembly/recombination methods. In addition to loading drugs into ferritin cages, therapeutic drugs are modified on the surface of ferritin to carry drugs. For example, the PD‐L1 binding peptide (PD‐L1pep1) was linked to the N‐terminus of HFn to block the interaction between PD‐1 and PD‐L1 for immunotherapy.^25^ Therefore, ferritin can be an excellent drug carrier for cancer treatment. The details are summarized in Table 1 and Figure 1.

## 3 FERRITIN FOR CANCER CHEMOTHERAPY

Chemotherapy is a common method for treating cancers, but the efficacy of chemotherapy is often limited due to the low targeting of chemotherapeutic drugs and the development of drug resistance in tumor cells.^69^ Ferritin nanocages have been widely used in the targeted delivery of therapeutic drugs due to their unique nanocage structure, excellent biocompatibility, intrinsic tumor targeting and good in vivo pharmacokinetics.^15^ We will elucidate this aspect in terms of efficacy improvement and toxicity control of drug‐loaded ferritin in the following part.

### 3.1 Improving the efficacy of chemotherapy

The chemical and physical characters of ferritin could be changed by surface modifications, and studies have demonstrated that additional functions could be introduced for drug delivery. For example, the ferritin drug carrier has dual‐targeting function by modifying the epidermal growth factor receptor (EGFR) targeting peptide to the surface of ferritin.^70^ Currently, researchers can modify ferritin by introducing different functional peptides into the surface of ferritin by genetic engineering, or by linking chemical groups to lysine and cysteine residues on the surface of ferritin.^23^, ^71^ This idea significantly improved the targetable potential of ferritin to tumor cells with low TfR1 level. In addition, methods for improving tumor infiltration capacity, cross the blood–brain barrier (BBB), enhance half‐life of ferritin nanocarriers have also been used already. These advanced modification methods for improving drug delivery efficacy of ferritin nanocages are being introduced in the following and summarized in Table 2.

**TABLE 2:** Modifications for increased efficacy.

Type of ferritin	Site	Surface modification	Effect	References
Human HFn	Surface and interior modifications	EGFR‐targeting peptides	Improve tumor targeting ability	40
Human HFn	Surface modification	RGD peptide	Improve tumor targeting ability	41
Horse spleen apoferritin	Surface modification	Folic acid	Improve tumor targeting ability	72
Human HFn	Surface modification	RGE peptide	Improve tumor targeting ability	59
Rat heavy chain ferritin	Surface modification	CGKRK peptide	Improve tumor targeting ability	53
Pyrococcus furiosus ferritin	Surface modification	SP94	Improve tumor targeting ability	44
Human HFn	Surface modification	Poly(ethylene glycol) modified	Improve tumor infiltration ability	49
Human HFn	Surface modification	Collagenase nanocapsules	Improve tumor infiltration ability	50
Short ferritin	Surface modification	Fibrin clot‐targeting peptides and microplasmin (μP) proteins	Improve tumor infiltration ability	51
Human HFn	Surface modification	Tumor‐penetrating peptide tLyP‐1	Improve tumor targeting and tumor infiltration ability	42
Human HFn	Surface modification	tumor‐penetrating peptide RGERPPR	Improve tumor targeting and tumor infiltration ability	73
Horse spleen apoferritin	Surface modification	GKRK peptide	Improve tumor targeting and ability to cross the BBB	43
Human HFn	Surface modification	Sequence DGAGGGEA	Improved drug loading and ability to cross the BBB	45
Rat heavy chain ferritin	Surface modification	scFv	Targeting cancer‐associated fibroblasts	62
Human HFn	Surface modification	PAS sequence	Improve half‐life of ferritin nanocarriers	46
Human HFn	Surface modification	PASE sequence	Improve half‐life and prevents ferritin from interacting with TfR1 in normal cells	74
Human HFn	Interior modification	PAS sequence and tumor penetrating peptide RGDK	Improve half‐life and tumor targeting	48
Human HFn	Interior modification	P13 hydrophobic peptide	Synergistically loading hydrophobic/hydrophilic drugs	39
Human HFn	Interior modification	Lysine or arginine	Increased load capacity for negatively charged nucleic acids	21

Abbreviations: BBB, blood brain barrier; DGAGGGEA, integrin α2β1 targeting peptide sequence; EGFR, epidermal growth factor receptor; GKRK, peptide ligand of heparan sulfate proteoglycan (HSPG); PAS, polypeptide sequences rich in proline (P), serine (S) and alanine (a) residues; PASE, insertion of two glutamic acid residues in the PAS sequence (E); RGD, three amino acid sequences targeting integrin Rvβ3; RGE, glioma targeting peptide; RGDK, integrin αvβ5‐targeted peptide; scFv, single‐chain variable fragment of fibroblast activation protein; SP94, targeting peptides for hepatocellular carcinoma biomarker GRP78; tLyP‐1, glioma targeting peptide.

Due to differences in the expression level of TfR1 on different tumor cells and the presence of the BBB, the efficacy of a single TfR1‐targeting ferritin drug carrier is limited.^70^ Therefore, researchers have improved the efficacy of ferritin drug carriers by targeting modifications to enable ferritin to obtain dual‐targeting functions. Integrin Rvβ3 is a biomarker of tumor angiogenesis and is expressed on the cell membrane of many tumor cells. RGD4C targeting peptide‐modified ferritin could efficiently target tumors for drug delivery by targeting integrins Rvβ3 and TfR1.^41^ Tumor‐penetrating peptides can improve the tumor infiltration of chemotherapeutic drugs and enhance anti‐tumor efficacy by targeting neuropilin‐1, which is overexpressed in tumor cells.^73^ Ma et al.^42^ modified the penetrating peptide tLyP‐1 to the N‐terminus of HFn by genetic engineering and loaded paclitaxel (PTX) into HFn nanocages. Animal experiments have shown that ferritin functionalized with penetrating peptides has dual receptor‐mediated targeting function and better interstitial infiltration efficiency. This modification transported the drug to deeper tumor areas, which was a good solution to the problem of insufficient drug delivery in solid tumors. In addition, the dual targeting function can also improve the ability of ferritin to cross the BBB, increasing the amount of drug reaching the brain parenchyma. Zhai et al.^43^ linked the GKRK peptide to ferritin via a sulfhydryl‐maleimide coupling reaction and encapsulated vincristine in a ferritin cage. GKRK peptides specifically target heparan sulfate proteoglycan overexpressed in angiogenesis and glioma. Experiments showed that the vincristine concentration in the brain of the modified drug carrier was 1.4 times that of the unmodified ferritin drug carrier. For the therapeutic effect, newly prepared ferritin nanocarriers treatment significantly prolonged the median survival of glioma mice compared with free vincristine. In addition to targeting peptides, folic acid can also be modified on the surface of ferritin to obtain dual targeting functions. Because cancer cells need to consume more vitamins, such as folic acid, during the proliferation period. So, folate receptors are overexpressed on the surface of many cancer cells compared to normal cells.^75^ Kashanian et al.^72^ designed a drug delivery system for epirubicin by modifying folic acid on the surface of horse spleen apoferritin. The obtained dual targeting ability could increase the affinity of the ferritin drug carrier to tumors, which reduced its side effects on normal cells. The antitumor results showed that the growth of MCF‐7 cells was significantly reduced by the newly prepared nanocarriers compared with free drugs.

Based on the targeting capacity of ferritin, the drug can be accurately released into the tumor area, which significantly improves the body's drug tolerance. Therefore, maximizing the quantity of loaded drugs of ferritin can extremely enhance the anticancer efficacy. Jiang et al.^44^ took advantage of the ultra‐high dose DOX loading capacity of Pyrococcus furiosus ferritin Fn to load 400 DOX drug molecules into Pyrococcus furiosus ferritin Fn. Combined with the GRP78‐targeted peptide SP94, a hepatocellular carcinoma biomarker, the modified ferritin was significantly infiltrated into the primary tumor, and killed the subcutaneous and lung metastatic tumors. Targeted modification of ferritin can also enhance the drug loading of ferritin. Huang et al.^45^ modified the targeting sequence DGAGGGEA of integrin α2β1 to the N‐terminus of HFn by genetic modification to prepare the 2D‐HFn. The drug encapsulation efficiency of the modified 2D‐HFn was as high as 61.7%, which was more than 5 times that of HFn. The drug loading capacity of each ferritin cage could even reach 458. Moreover, the BBB penetration ability of 2D‐HFn was 2 times that of HFn.

It should be noted that due to the smaller particle size of ferritin, its plasma half‐life is short (approximately 2 h).^15^ To address this shortcoming, Falvo et al.^46^ fused sequences rich in proline (P), serine (S), and alanine (a) residues (PAS) to the N‐terminal subunit of HFn by genetic engineering, producing HFn‐PAS. The data showed that the half‐life of DOX‐loaded HFn‐PAS (15.6 ± 0.55 h) was significantly longer than that of DOX‐loaded HFn (2.7 ± 0.3 h). In another study, Falvo et al.^74^ linked human HFn to PAS sequences via a matrix metalloproteinase cleavable peptide. Then, HFn‐MP‐PASE was prepared by inserting two glutamic acid residues (E) into the PAS sequence by genetic engineering. HFn‐MP‐PASE prevented the interaction of HFn with normal cell surface TfR1 to prevent internalization into normal cells. When it entered the tumor microenvironment (TME), the highly expressed matrix metalloproteinase could cleave the peptide fragment, which allowed the PASE polypeptide to dissociate from the ferritin surface for therapeutic efficacy. On this basis, Falvo et al.^47^, ^76^ replaced four native HFn residues with glutamic acid residues to encapsulate a non‐camptothecin topoisomerase I inhibitor (Genz‐644282) in HFn‐MP‐PASE. This produced The‐05 with negatively enhanced inner surface of HFn. Then, Genz‐644282 was encapsulated in a ferritin cage to obtain The‐0504 by disassembly and recombination. In the The‐0504 treatment group, tumor growth inhibition rate and objective response rate of PaCa44, MDA‐MB‐231 and HepG2 tumor‐bearing mice were all reached 100%. In the treatment of colorectal cancer, mice treated with The‐0504 survived an experimental period of 100 days, which was significantly higher than the median survival (45 days) of mice treated with free Genz‐644282. On the basis that the PAS sequence is known to prolong the half‐life of ferritin. To achieve active targeting to tumor cells, Yin et al.^48^ fused PAS peptide and integrin αvβ5‐targeted peptide RGDK together to the C‐terminal subunit of HFn. The results showed that the half‐life of HFn modified with both peptides increased by 5‐fold compared to unmodified HFn, which significantly increased the amount of drug accumulated in the tumor area.

Although the targeting and half‐life of ferritin nanoparticles can be enhanced by surface modification of ferritin, the abundance of hyaluronic acid, fibrin, collagen, and other extracellular matrix (ECM)‐related molecules around tumor cells prevented ferritin infiltration to a great extent.^77^ By modifying ferritin, researchers have enhanced the ability of ferritin nanoparticles to cross the ECM and tumor vascular density. Comerford et al.^49^ modified poly ethylene glycol on the surface of ferritin nanocages. Poly ethylene glycol‐modified HFn could reduce the interaction with ECM components in tumor tissue and enhance its ability to cross the tumor stroma. Afterwards, the HIF1α inhibitor acriflavine (AF) was loaded into ferritin and transported to the hypoxic tumor site. AF can inhibit HIF‐1α‐mediated up‐regulation of multidrug resistance protein 1 to restore chemotherapy efficacy. AF‐loaded ferritin nanoparticles in combination with cisplatin significantly inhibited tumors in mice. Yao et al.^50^ linked collagenase nanocapsules to HFn via amide bonds and loaded DOX into HFn. Collagenase could enhance the tumor permeability of ferritin nanoparticles by hydrolyzing collagen in the ECM. Experiments showed that treatment with HFn modified with collagenase nanocapsules significantly increased tumor vascular density after 24 h of treatment, while DOX‐loaded HFn alone had no change in tumor vascular density. Due to the increased vascular density, the accumulation and distribution of ferritin nanocarriers in the tumor would also increase. Fluorescence imaging also showed that the HFn modified with collagenase nanocapsules was distributed in almost all tumor extents. In the 4T1 breast cancer animal model, newly prepared ferritin nanocarriers achieved an 85% tumor inhibition rate. Seo et al.^51^ linked fibrin clot‐targeting peptide CLT and microplasmin μP proteins to short ferritin cages for preparing fibrinolytic nanocages. Fibrinolytic nanocages could target clots in the TME through the action of CLT and dissolve clots through μP. Fibrin deposition was significantly reduced in mice treated with fibrinolytic nanocages compared to CLT or μP alone. Furthermore, the fibrin deposits in the tumor blood vessels were completely cleared. This could reduce the risk of venous thromboembolism in cancer patients and facilitate drug delivery to increase drug distribution in the tumor. In B16F10 melanoma tumor‐bearing mice, tumors co‐administered with fibrinolytic nanocages and DOX exhibited a broader distribution of DOX‐related fluorescence than DOX alone.

### 3.2 Reducing chemotherapy toxicity

**FIGURE 2:**
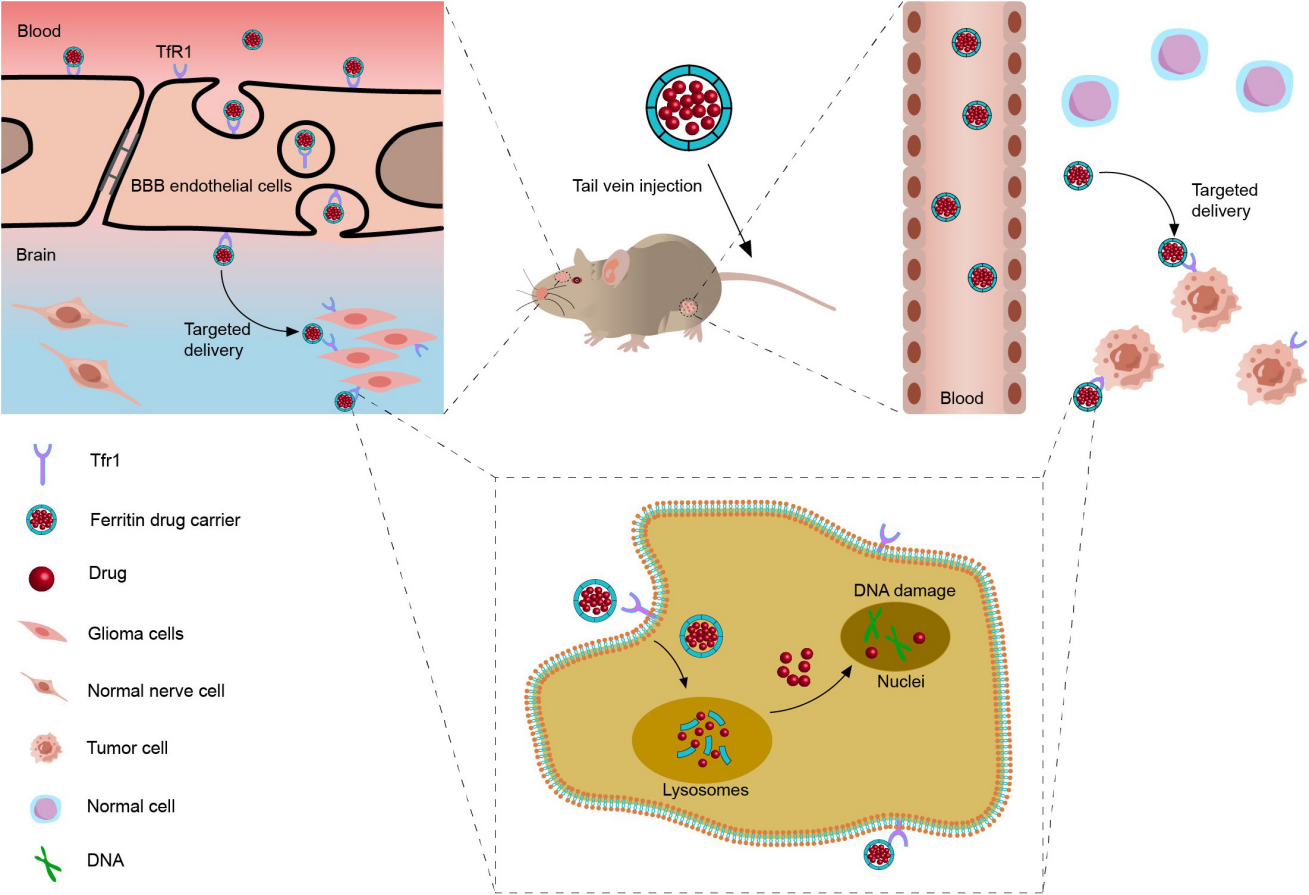
Ferritin for drug delivery. Ferritin drug carriers can target tumor cells and enter cells through receptor‐mediated internalization. In addition, ferritin can also cross the blood–brain barrier to deliver drugs to gliomas. After the ferritin drug carrier enters the tumor cells, it can be degraded in the lysosome and release the drug to kill the tumor cells.

In chemotherapy, drug delivery via ferritin significantly reduces drug systemic toxicity. DOX‐loaded human HFn could deliver DOX to tumor sites by targeting TfR1. Subsequently, HFn was internalized into tumor cells by interacting with overexpressed TfR1, and the carried DOX was released into tumor cells via lysosomes. Importantly, due to the tumor targeting of ferritin, the HFn nanocages could significantly reduce DOX concentrations in healthy organs of mice, which resulted in a 4‐fold increase in maximum tolerated dosing.^15^ In addition, DOX‐loaded HFn could also cross the BBB. Human HFn could target highly expressed TfR1 in BBB endothelial cells and cross the BBB by vesicular transport (Figure 2), which enabled targeted therapy for gliomas and addressed the limitation that chemotherapeutics cannot cross the blood–brain barrier.^37^ Studies have shown that there was a high affinity between PTX and HFn. The encapsulation efficiency of PTX‐loaded HFn was higher than that of DOX, curcumin, and Olaparib.^38^ Liu et al.^38^ loaded PTX into HFn for the treatment of glioma. Due to the high stability of ferritin, the amount of drug released during drug transport was very small. Only when ferritin nanoparticles enter the lysosomes of tumor cells would the drug be decomposed and released. Compared with PTX alone, the use of HFn could significantly increase PTX concentrations in brain tumors and decrease PTX concentrations in normal tissues. Therefore, based on the tumor targeting of ferritin, ferritin drug carriers could enter tumor cells through receptor‐mediated internalization and release drugs in lysosomes, which could significantly reduce the side effects of chemotherapy drugs (Figure 2). Furthermore, Moon et al.^40^ constructed a non‐toxic drug delivery platform based on HFn, which inactivated chemotherapeutic drugs during delivery and activated them in tumor cells. They modified the N‐ and C‐termini of the HFn subunit by gene insertion of the core functional peptide to construct a subunit mutant NH2‐YHWYGYTPQNVI (insert 1)‐huhf subunit‐h6‐linked‐gspafla (insert 2)‐(DE)_3_(insert 3)‐COOH. Insert 1 was a peptide specifically targeting epidermal growth factor receptor (EGFR), Insert 2 was a sequence specifically recognized and cleaved by the endosomal protease CTSE, and Insert 3 was a carboxyl‐rich peptide (DE)_3_. By the carboxyl group, (DE)_3_ could be covalently bound to GEM, MMC and DOX with amine groups. In addition, the overexpressed endosomal protease CTSE in tumor cells could cleave the insert 3 sequence to release and activate chemotherapeutic drugs. Experiments showed that in normal cells without CTSE, the drug was difficult to release from the freshly prepared ferritin drug platform and thus remained inactive. Therefore, only when the ferritin drug carrier entered the tumor cells would the chemotherapeutic drugs be released and exerted anti‐tumor effects. This approach further reduced the damage to healthy organs from chemotherapy drugs.

Thus, ferritin drug carriers deliver chemotherapeutic drugs to tumor tissues by targeting TfR1 highly expressed in tumor cells, and can also cross the BBB by targeting TfR1. This increases the drug's accumulation in the tumor area and reduces its accumulation in normal tissues. Therefore, the use of ferritin drug carriers significantly improves antitumor efficacy and reduces systemic side effects. In addition, the tumor targeting, drug loading and plasma half‐life of ferritin can be improved by surface modification of ferritin. Importantly, the modified ferritin nanocarriers can disorganize the TME, relieve tumor hypoxia circumstance, and promote the tumor infiltration capacity of drugs.

## 4 FERRITIN FOR PHOTODYNAMIC THERAPY AND PHOTOTHERMAL THERAPY

**FIGURE 3:**
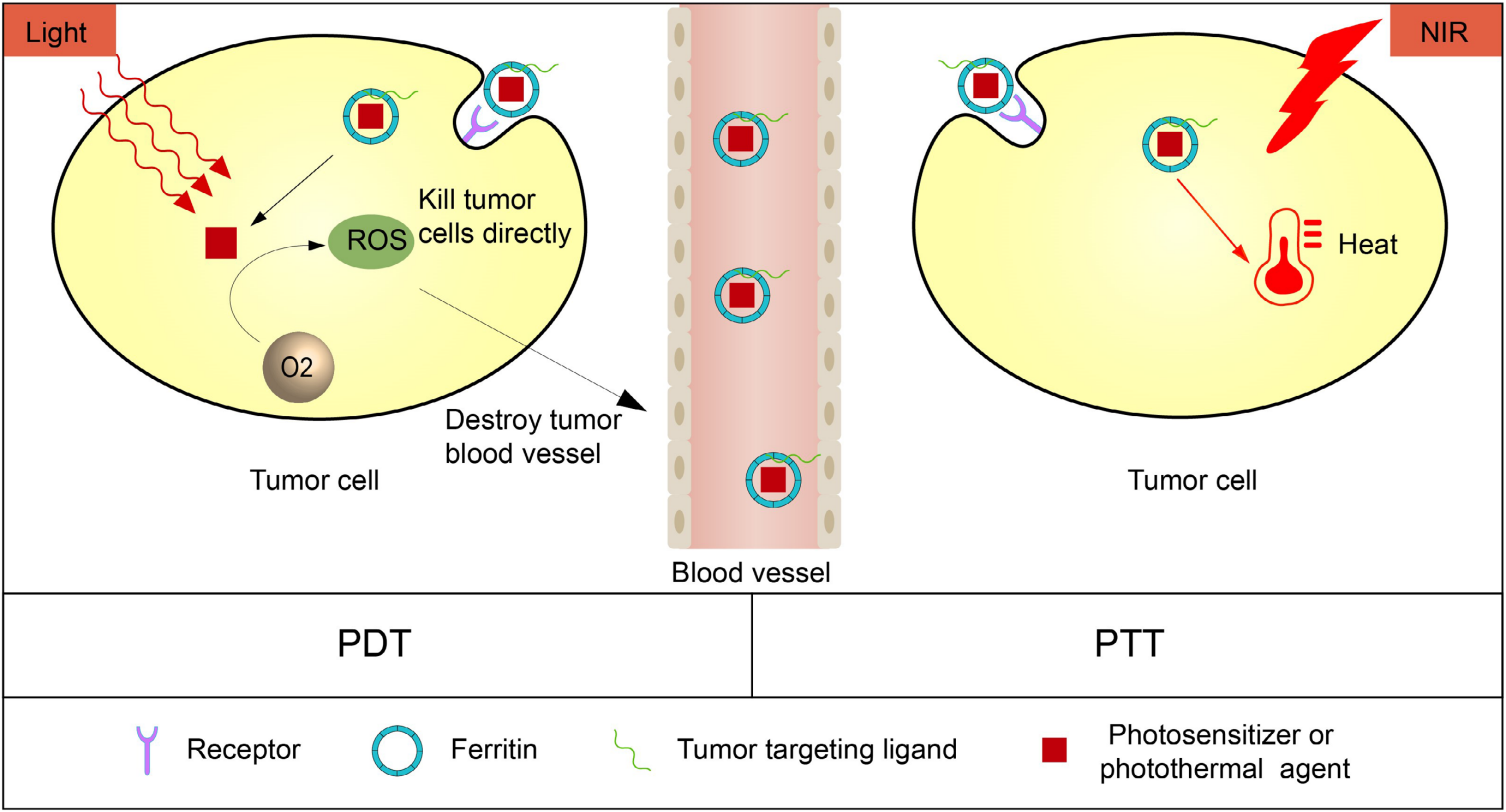
Ferritin for photodynamic therapy (PDT) and photothermal therapy (PTT). Ferritin nanoparticles can deliver photosensitizers and photothermal agents to tumor cells for precise PTT and PDT of tumor cells. Ferritin transports the photosensitizer to tumor cells, and by applying a specific wavelength of laser light, the photosensitizer produces reactive oxygen species in the presence of oxygen. PDT can directly exert cytotoxic effects on cancer cells, or destroy tumor blood vessels, as well as stimulate anti‐tumor immunity. Ferritin transports photothermal agents into tumor cells to induce PTT, which destroy tumor cells or tissues by absorbing near‐infrared light and converting it into heat.

Photodynamic therapy (PDT) treats tumors by applying a specific wavelength of laser light to cause photosensitizers to generate reactive oxygen species, such as singlet oxygen ^1^O_2_, in the presence of oxygen^78^ (Figure 3). PDT can directly exert cytotoxic effects on cancer cells, or destroy tumor blood vessels, and stimulate anti‐tumor immunity.^78^ However, targeted delivery of photosensitizers to tumors and retention of photosensitizers at tumor sites are still problems to be solved. Additionally, PDT requires a photosensitizer, oxygen, and a specific wavelength of laser light.^79^ However, hypoxia is a prominent feature of solid malignancies,^80^ which induces tumor resistance to PDT. In a recent study, Zhu et al.^22^ loaded MnO_2_ into ferritin via biomineralization, and then the photosensitizer chloride e6 (Ce6) was captured into the HFn cage. Insufficient intratumoral blood supply and increased cellular metabolism could result in acidification of the TME and increase intratumoral H_2_O_2_. Moreover, MnO_2_ could react with H_2_O_2_ to generate oxygen in an acidic environment to improve the hypoxic TME and increase the photodynamic efficacy. Therefore, HFn loaded with MnO_2_ and Ce6 had a good effect in overcoming tumor hypoxia and enhancing the photodynamic effect of tumor therapy. In the 4T1 mouse tumor model, fluorescence imaging showed that the fluorescence signal of HFn loaded with MnO_2_ and Ce6 in the tumor gradually increased over time. Moreover, the retention time in the tumor is also longer than that of free chloride e6. Among the anti‐tumor effects, the HFn loaded with MnO_2_ and Ce6+ laser irradiation group had stronger anti‐tumor effect than free Ce6+ laser irradiation. Therefore, ferritin nanoparticles can target the photosensitizer to the tumor site to increase the therapeutic effect. Ferritin‐based PDT can also selectively kill cancer‐associated fibroblasts, which is an important component of TME. Li et al.^62^ loaded the photosensitizer ZnF16pc into rat HFn by a pH‐based disassembly /recombination method. At the same time, the single‐chain variable fragment (scFv) of fibroblast activation protein was modified on the surface of the ferritin cage. Newly prepared ferritin nanocarriers could specifically target fibroblast activation protein expressed in cancer‐associated fibroblasts (CAF). In the 4T1 mouse tumor model, PDT guided by ferritin nanocarriers could significantly decompose CAF‐mediated ECM. Immunofluorescence staining showed that PDT treatment increased tumor uptake of the relatively large nanoparticles by more than five‐fold. This suggested that the modified ferritin introduced drug delivery could also significantly enhance the treatment effects of PDT therapy in another term of eliminating tumor ECM‐mediated drug resistance.

Photothermal therapy (PTT) is a method in which photothermal agents destroy tumor cells or tissue by absorbing near‐infrared light and converting it into heat.^81^ In general, when tissue temperatures greater than 60°C are achieved by PTT, cells die almost instantaneously because of protein denaturation and plasma membrane disruption^79^ (Figure 3). However, since the lack of targeting of photothermal agents can damage normal tissues, ferritin as a carrier of photothermal therapy will increase the efficacy of PTT and reduce side effects. Zhang et al.^53^ modified CGKRK peptide at the N‐terminus of mouse HFn. The HFn modified with CGKRK peptide could target heparan sulfate highly expressed on the surface of neovascular endothelial cells and tumor cells. Afterwards, the near‐infrared absorbing organometallic aromatic complex 556‐Ph could be loaded into HFn by a pH disassembly/reassembly‐based approach. The 556‐Ph could not only exhibit heating effect under 808 nm laser irradiation, but also generated reactive oxygen species under 630 nm laser irradiation. Therefore, HFn loaded with 556‐Ph had the effect of combining PDT and PTT. In the MDA‐MB‐435 mouse tumor model, PDT combined with PTT treatment completely cleared the tumor without recurrence after 16 days.

In addition, PTT could also combine with chemotherapy by means of modified ferritin nanocarrier. Li et al.^55^ coupled the second near‐infrared region dye IR1061 with the tumor target molecule Folic acid (FA) on the surface of horse spleen apoferritin (AFN). Afterwards, an “all‐in‐one” nanoplatform (IR‐AFN@PTX‐FA) for photothermal therapy and chemotherapy was prepared by loading PTXs into AFN through a PH‐mediated disassembly/reorganization approach. Under irradiation at a wavelength of 1064 nm, the tumor was synergistically treated by laser irradiation and PH induction to generate heat and release PTX at the tumor site, respectively. Because the 1064 nm wavelength laser has deeper tissue penetration and generates a higher temperature than the 808 nm laser, IR‐AFN@PTX‐FA is suitable for large solid tumors or deep tissue tumors. In vivo experiments showed that IR‐AFN@PTX‐FA could significantly inhibit tumor growth in a mouse model of breast cancer. Based on the high packaging capability of ferritin nanocage, superior material harbor dual structure was constructed and showed unexpected treatment effect. Guo et al.^54^ developed a drug dual‐carriers delivery system (DDDS) by nanoscale graphene oxide, which had large surface area, good drug loading rate and near‐infrared photothermal effect. Firstly, the nanoscale graphene oxide was bound to the mitochondria‐specific target molecule IR780 and loaded with the anticancer drug resveratrol (RSV) to form a nanocomplex IR780‐NGO‐RSV. Afterwards, IR780‐NGO‐RSV were loaded into ferritin by a PH‐mediated disassembly/reassembly method. Experiments showed that DDDS first localized to the lysosome of tumor cells to dissolve ferritin to release IR780‐NGO‐RSV. After that, under near‐infrared light irradiation, the mitochondria‐targeted IR780‐NGO‐RSV could induce RSV release based on heat. In vivo experiments showed that DDDS had the efficacy of chemotherapy combined with photothermal therapy. After 2 months of initial treatment, DDDS‐treated mice had a 100% survival rate. Moreover, to improve transport efficiency of ferritin itself for PTT, specific crust was constructed. Wang et al.^56^ mixed HFn with calcium chloride in Dulbecco's modified Eagle's medium. The acidic amino acid residues on the surface of HFn could chelate calcium ions (Ca^2+^) and provide nucleation sites to generate tiny crystals, promoting in situ biomineralization. Calcium phosphate shells formed on the outside of the ferritin cage as the tiny crystals gradually grew and densified. Calcium phosphate shell could prevent ferritin from binding to the highly expressed ferritin receptor in the liver and would be soluble in the acidic TME. Therefore, the calcium phosphate shell could prevent the hepatic entrapment of ferritin without affecting drug release. Wang et al. loaded arsenic trioxide into ferritin and loaded indocyanine green onto ferritin nanoparticles through the interaction of negatively charged sulfonic acid groups in indocyanine green with Ca^2+^. This combined chemotherapy and PTT. Animal experiments demonstrated that this method could completely suppress advanced HeLa mouse tumors with a volume of 300 mm^3^. In the MCF‐7 and MGC‐803 mouse models, the tumor volume was also suppressed to less than 150 mm^3^ after 26 days of treatment.

The above studies show that by loading or linking photothermal agents and photosensitizers, ferritin is a promising nanocarrier for tumor PTT, PDT and synergistic therapies.

## 5 FERRITIN FOR IMMUNOTHERAPY

Immunotherapy is aimed at boosting autoimmunity to eliminate malignant tumors. In recent years, ferritin has emerged as an attractive nanoparticle for immunotherapy. Modified ferritin nanocarriers could not only boost antigen presentation and promote the infiltration of immune cells, but also enhance the killer effect of immune cells.

### 5.1 Priming the immune system

**FIGURE 4:**
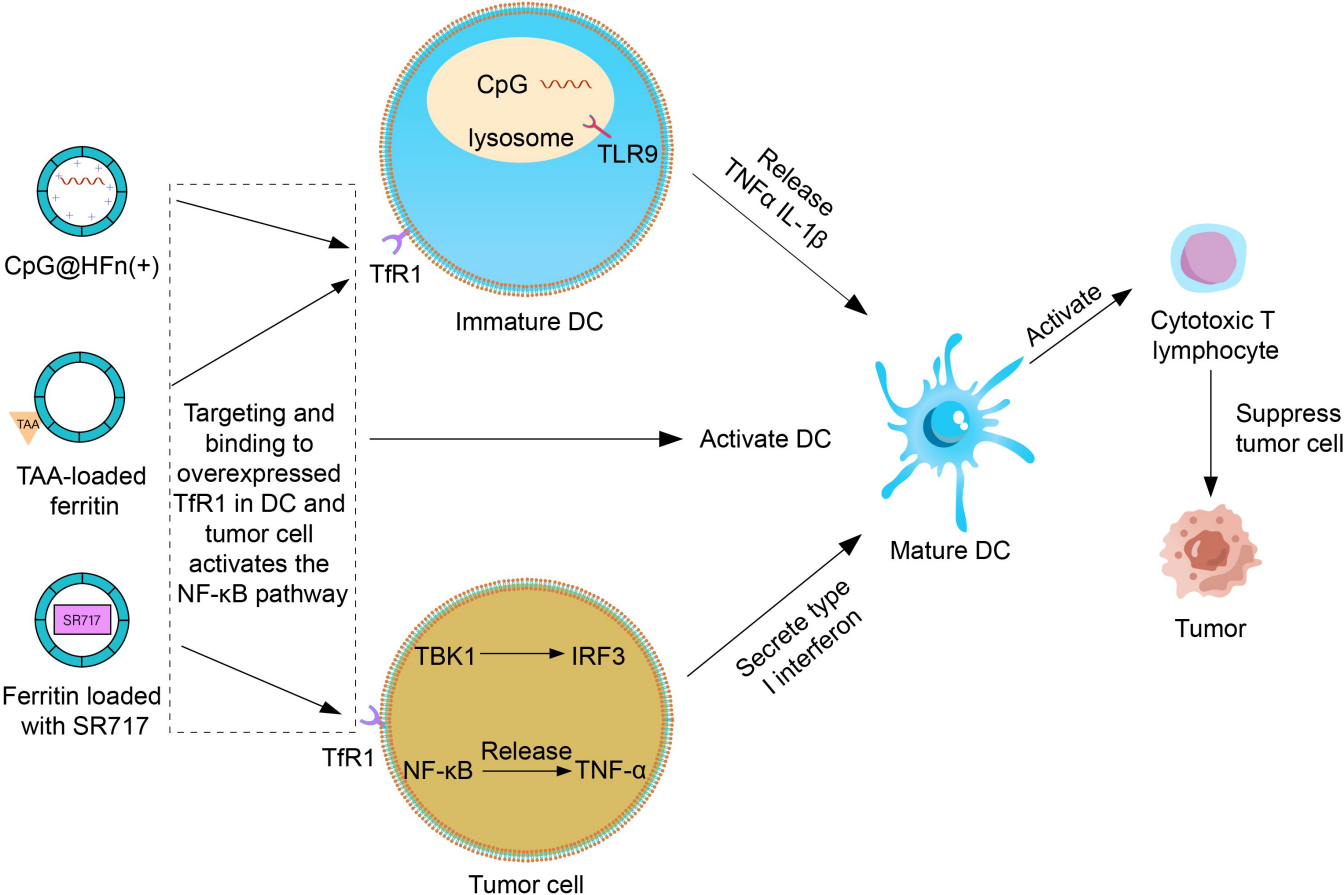
Ferritin for immune activation. Ferritin can target delivery of antigens, nucleic acids, and adenosine monophosphate analogs to enhance immune efficacy to ameliorate the above limitations of immune activators. The N‐terminal region of the heavy‐chain subunit a‐helix is directly involved in HFn binding to TfR1, which can mature DC to trigger T cell responses through hyper‐activation of NF‐κB signaling pathway. The interaction of CpG with TLR9 in DC can activate the NF‐κB pathway. This enables DC to release pro‐inflammatory cytokines, such as TNF‐α and IL‐1β, and triggers DC maturation to enhance their antigen‐presenting capacity. Upon activation, STING recruits and activates tank‐binding kinase 1 (TBK1), which phosphorylates the transcription factor IRF3 to induce increased secretion of type I interferons. Type I interferons can activate DC to promote the priming of cytotoxic T cells. In addition, the activation of the STING pathway also leads to the production of inflammatory cytokines (e.g., TNF‐α) through the NF‐κB pathway.

Ferritin nanocarriers primarily activate the immune system by enhancing antigen presentation and activating immune cells. First, ferritin nanocarriers can be loaded with antigens to promote the maturation of antigen‐presenting cells for immune activation. Dendritic cells (DCs) are known to activate T cells to generate cytotoxic T cells when they take up tumor‐associated antigens in LNs to treat tumors^82^ (Figure 4). However, the ability of therapeutic antigens to target antigen presenting cells is weak, and antigens that do not reach lymph nodes (LNs) are rapidly cleared and ignored by the immune system.^83^ By comparing the ability of four different protein nanoparticles in targeting LNs, Lee et al.^57^ found that human HFn could rapidly target LNs and accumulate sufficiently in LNs. This rapid cumulative effect was due to the strong binding of HFn to T cell immunoglobulin and mucin domain‐2 present in LNs. Afterwards, the tumor‐specific antigen red fluorescence protein (RFP) was genetically fused to the C‐terminus of HFn. RFP‐modified HFn targeted LNs and induced strong antigen‐specific CD8+ T cell responses, successfully inhibiting tumor growth in tumor‐bearing mice. In addition, HFn can also target overexpressed TfR1 in DCs to be endocytosed by DCs. The N‐terminal region of the heavy‐chain subunit a‐helix is directly involved in HFn binding to TfR1, which can mature DCs to trigger T cell responses through hyper‐activation of NF‐κB signaling pathway^84^, ^85^ (Figure 4). However, affected by various factors, somatic cells mutate to generate tumor neoantigens and present them on tumor cells.^86^ For the presentation of neoantigens, protein modification steps need to be performed again, which cannot provide personalized tumor immunotherapy. SpyTag/SpyCather combination technology can establish an efficient ferritin antigen presentation platform. SpyTag and SpyCatcher can be rapidly bound together by a covalent bond under almost any condition.^87^ Wang et al.^88^ fused SpyCatcher to the N‐terminus of ferritin by genetic engineering for preparing SpyCatcher‐ferritin nanoparticles. SpyCatcher‐ferritin could bind to antigens containing the SpyTag gene. Compared with soluble peptide antigens, SpyCatcher‐ferritin nanoparticles carrying MC38 tumor‐derived mutated neoantigen enhanced cytotoxic T‐lymphocyte response 2‐3‐fold and significantly inhibited tumor growth.

In addition, ferritin nanocarriers can also be loaded with nucleic acids or adenosine monophosphate analogs to activate immune cells. Toll‐like receptor 9 (TLR9) agonists have been shown to have great potential as immune adjuvants, and the interaction of CpG with TLR9 in DCs could activate the nuclear factor‐kappaB (NF‐κB) pathway. This enabled DCs to release pro‐inflammatory cytokines, such as tumor necrosis factor alpha (TNF‐α) and interleukin 1β (IL‐1β), and triggered DC maturation to enhance their antigen‐presenting capacity.^89^, ^90^ However, free nucleic acids cannot penetrate cell membranes and are easily removed by nucleases, so the single in vivo delivery efficiency of nucleic acids is not ideal. Zhang et al.^21^ constructed an internally positively charged ferritin (HFn(+)) by replacing negatively charged glutamic acid and aspartic acid residues with positively charged lysine or arginine in the inner cavity of HFn. This could enhance the loading capacity of ferritin for negatively charged nucleic acids. Afterwards, CpG were encapsulated in HFn(+) by a pH‐mediated disassembly /recombination method. CpG @HFn(+) encapsulating CpG could enter DCs and release CpG under the action of lysosomes. Afterwards, CpG could recognize and interact with TLR9 on the lysosomal membrane to enhance the antigen‐presenting ability of DCs and promote immune activation. In addition, they also linked the photosensitizer Ce6 to the HFn(+) surface via covalent bonding of amino and carboxyl groups. Immunogenic cell death of tumor cells could be induced by PDT. Mechanistically, PDT‐induced ICD could promote the release of tumor‐associated antigens which would be captured by DCs. Therefore, ferritin nanocarriers could combine CpG‐induced enhancement of DC antigen presentation and PDT‐induced TAA release for T cell activation (Figure 4). In the 4T1 tumor mouse model, tumors were completely eliminated in most mice treated with PDT combined with Ce6‐CpG@HFn (+). In addition, CpG oligonucleotides could also induce polarization of tumor‐associated macrophages (TAM). In the TME, the most abundant immune cells are TAM, which can be divided into anti‐tumor M1 type and tumor‐promoting M2 type.^91^ However, in the TME, TAM are often M2‐type macrophages to support tumor growth.^92^ Therefore, polarizing M2‐type macrophages to M1‐type is a strategy to suppress cancer. Shan et al.^58^ genetically engineered an M2‐type macrophage targeting peptide to the N‐terminus of human HFn and loaded CpG into human HFn. The newly prepared nanocarriers could target M2‐type macrophages in the TME to repolarize them to M1‐type. M1‐type macrophages could secrete pro‐inflammatory cytokines and chemokines (e.g., IL‐6, IL‐12, and TNF‐α) for antigen presentation and tumor cell clearance. In vivo antitumor experiments showed that the newly prepared ferritin nanocarriers could significantly inhibit tumor growth in mice, which might be due to the significant increase in the M1/M2 ratio in the tumors.

Stimulator of interferon genes (STING) is an important immune‐related antitumor molecule through type I interferons.^93^ But STING is expressed in both tumor cells and normal cells, so the use of STING agonists can trigger systemic inflammation.^94^ Based on the tumor‐targeting properties of ferritin, STING agonists can be delivered into tumor cells. Wang et al.^59^ modified the tumor‐targeting peptide RGE at the N‐terminus of HFn by genetic engineering, which improved the ability of ferritin to target tumors and cross the BBB. Afterwards, the STING agonist SR‐717 was loaded into HFn by disassembly and recombination method to prepare SR‐717@RGE‐HFn nanoparticles. In a mouse glioma model, the mRNA expression of IL‐β1 and TNF‐α in tumor cells was significantly increased by SR‐717@RGE‐HFn treatment. The number of CD8+ T effector cells in the TME was also 9.56 times higher than in mice treated with SR‐717 alone. In conclusion, ferritins can carry antigens, nucleic acids, and adenosine monophosphate analogs to boost immune responses, which ameliorate their limitations for tumor immunotherapy (Figure 4).

### 5.2 Enhance treatment effect by blocking immune checkpoint

By antagonizing the interaction between receptors and ligands, ferritin provides a new approach to immune checkpoint inhibition therapy. Among all immune checkpoints, PD‐1/PD‐L1 remains the most widely used one. Thus, modified ferritins interfering this signal also showed compelling treatment effects. Kim et al.^61^ integrated the extracellular domain gene of PD‐1 into the C‐terminus of HFn to obtain newly prepared ferritin nanocarrier PDNC, which was 1000‐fold higher in affinity for PD‐L1 compared with PD‐1. Based on the targeting of ferritin to tumor cells and LNs, PDNCs could rapidly accumulate in tumor‐draining lymph nodes. Experiments showed that PDNC in tumor‐draining lymph nodes effectively induced DC‐mediated T cell activation, increased CD8+ T cell infiltration in tumors, and resulted in 33% complete tumor regression. In another study, Jeon et al.^25^ combined chemotherapy and immunotherapy by linked the PD‐L1‐binding peptide (PD‐L1pep1) to the N‐terminus of HFn to construct a PpNF nanocages that could specifically bind to PD‐L1. PD‐L1pep1 could activate T cells by blocking the interaction between PD‐1 and PD‐L1. Compared with PD‐L1pep1, PpNF was 100 times more effective in activating T cells than PD‐L1pep1. Then, they loaded 130 DOX molecules into PpNF nanocages and injected them into tumor‐bearing mice. As a result, the inhibitory effect of DOX‐loaded PpNF was significantly better than that of PpNF, DOX and anti‐PD‐L1 antibody alone. In addition, as aforementioned in the PTD part, modified ferritin also exerted its effect through eliminating immunosuppressive cancer‐associated fibroblasts in the TME, and showed synergistic effect with PD‐L1 blockade.^62^, ^64^

**FIGURE 5:**
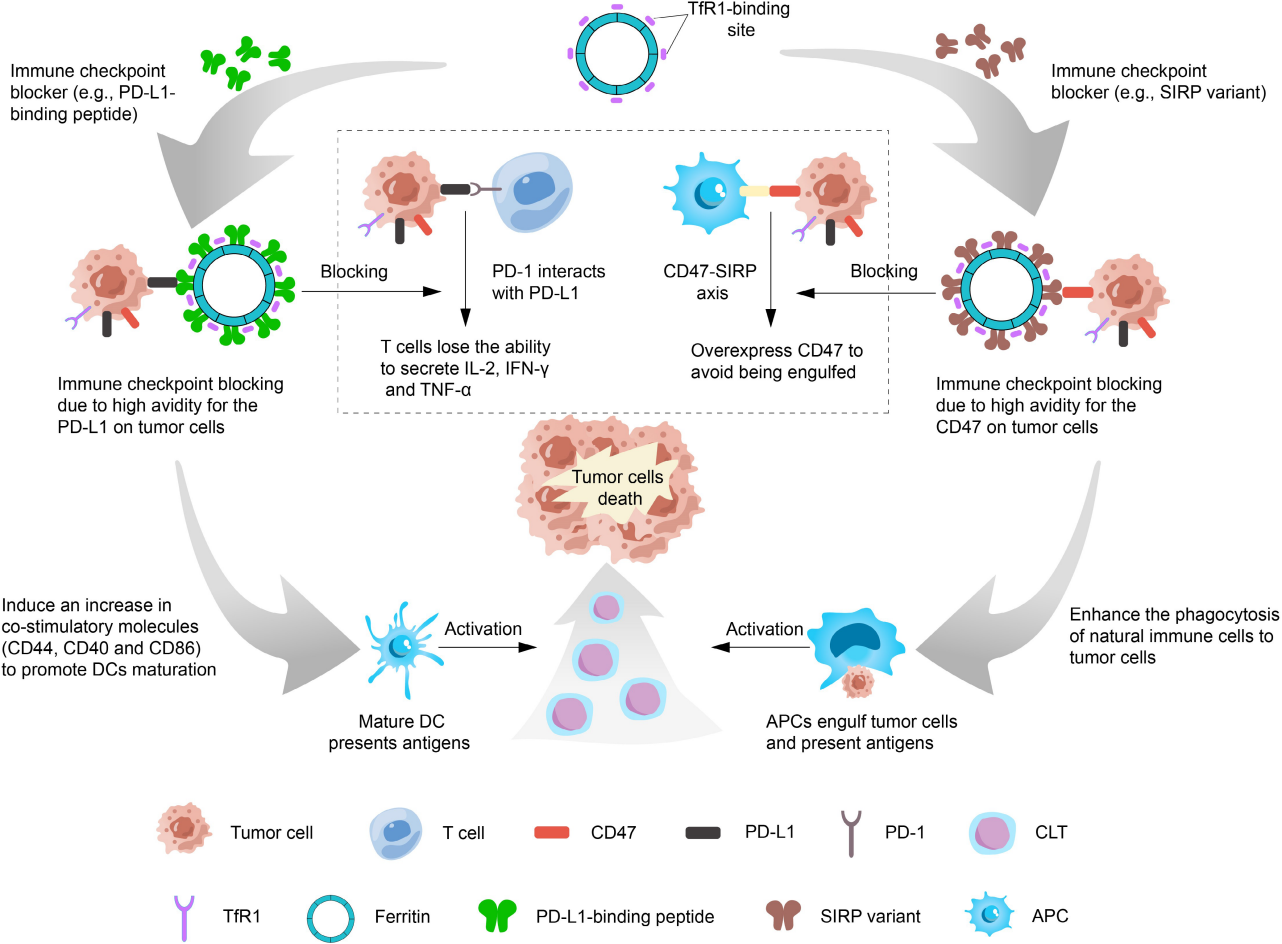
Ferritin for boosting immunity. The interaction of PD‐1 on the surface of T cells with PD‐L1 on the surface of tumor cells can cause T cells to lose the ability to secrete IL‐2, IFN‐γ, and TNF‐α and fail to eliminate tumor cells. T cell activation can be promoted by blocking the interaction between PD‐1 and PD‐L1. Ferritin modified with PD‐L1 binding‐peptide can inhibit immune checkpoints due to high avidity for PD‐L1. This induces an increase in co‐stimulatory molecules (CD40 and CD86) on DCs and CD44 in CD8+ T cells to promote dendritic cell maturation. In addition, tumor cells avoid phagocytosis by immune cells by upregulating CD47. Phagocytosis of tumor cells by immune cells can be increased by blocking the CD47‐SIRP axis. Ferritin modified with SIRP variant can inhibit immune checkpoints due to high avidity for CD47.

In addition, tumor cells avoid being engulfed by innate immune cells by overexpressing CD47, a kind of “don't eat me” signal.^95^ One study showed that blocking the CD47‐ signal regulatory proteins α (SIRPα) axis between tumor cells and phagocytes enhanced the phagocytosis of tumor cells by innate immune cells.^96^ Lee et al.^24^ used SIRPα variants to modify the surface of HFn to prepare FHSIRPα nanoparticles. FHSIRPα efficiently bound and antagonized CD47 in mice. In addition, Lee et al. combined chemotherapy with immunotherapy. Experiment showed that DOX‐loaded FHSIRPα‐DOX increased the percentage of T cell proliferation more than FHSIRPα alone. This indicated that FHSIRPα‐DOX enhanced APCs‐mediated phagocytosis. Afterwards, APCs could enhance the processing of tumor antigens and present them to antigen‐specific T cells. Among the antitumor effects, FHSIRPα‐DOX completely eradicated CT26 and B16 tumor cells in mice. In another study, Lee et al.^63^ synthesized an albumin‐binding peptide‐cysteine protease cleavable linker‐doxorubicin conjugate (MPD‐1). Albumin‐binding peptides could extend the half‐life of the drug to 106 hours. Afterwards, the release of caspase‐3 triggered by radiation (RT) cleaved the above complex and released the active drug DOX. Compared with DOX alone, MPD‐1+ RT treatment increased the number of CD4+ and CD8+ T cells and induced immunogenic death. At the same time, the combination of ferritin expressing SIRPα resulted in complete tumor eradication in 8 of the 9 mice. And the combination therapy also elicited tumor‐specific memory. When the same cancer cells were implanted on the other side of the cured mice, the mice were still able to suppress tumor growth. Besides SIRPα, the V1 domain of signal regulatory proteins γ (SIRPγ) has also been reported to bind to CD47.^97^ To further improve the blocking efficiency of CD47, Choi et al.^60^ coupled SIRPγ variants to the C‐terminus of human HFn for designing FSγV. In vitro experiments showed that FSγV led to a significant increase in the phagocytosis of B16F10 cells by macrophages and DCs. This could facilitate antigen presentation and mediate T cell priming and activation. Similar results for phagocytic activity, the proportion of mature DCs and antigen‐experienced CD8+ T cells in tumor‐draining lymph nodes was significantly increased in FSγV‐treated mice. In addition, Choi et al. also found that the combination of CpG and FSγV up‐regulated the phagocytosis of B16F10 cells by mouse dendritic cells and macrophages compared with FSγV alone. As described above, ferritin nanocarriers further increased the proportion of CD8+ T cells in mouse TME through a combination of immune priming systems and blocking immune checkpoints (Figure 5).

In conclusion, the study shows that ferritin nanoparticles are a promising carrier for immunotherapy and address the deficiencies of immunotherapy to a certain extent. Moreover, the immunogenic anti‐tumor efficacy can also be enhanced by combining chemotherapy, PTD, and PTT by loading specific drugs and materials.

## 6 CONCLUSIONS AND FUTURE PERSPECTIVES

Most existing cancer treatments lack tumor cell targeting ability, which is often ineffective and has many side effects. Ferritin‐based nanoplatforms have attracted widespread interests among researchers due to their excellent biocompatibility, abundant functional groups, and inherent tumor‐targeting properties. Ferritin specifically targets tumor cells that highly express the TfR1. By loading chemotherapeutic drugs into their own ferritin cages, chemotherapeutic drugs can be delivered to the tumor area for release and reduce the systemic toxicity of chemotherapeutic drugs. With further research on ferritin, researchers have improved the ability of ferritin for drug delivery through surface modifications. First, some new tumor markers were discovered by the researchers. Modification of the ferritin surface with targeting peptides can further enhance tumor targeting. Second, surface modification of ferritin can also increase the drug loading and half‐life of ferritin. The optimized ferritin nanocarriers can increase drug aggregation at tumor sites, which significantly improves the efficacy of chemotherapeutic drugs. Importantly, ferritin can present antigens or nucleic acids to promote the maturation of antigen‐presenting cells to activate immunity. Modified ferritins can also inhibit the interaction between receptors and ligands for immune checkpoint inhibitor therapy. Similarly, in both chemotherapy and immunotherapy, the ECM components of the TME limit therapeutic efficacy. However, modification of ferritin can disrupt ECM components outside tumor cells, which increases tumor infiltration of ferritin drug carriers and T cells. In addition, PTT and PDT are also promising field for ferritin application. In addition, ferritin nanoparticles have also realized the combined treatment of various therapeutic methods.

However, ferritin nanocarriers still have many limitations. First, drug loading based on disassembly/reassembly methods often leads to the formation of cavitation defects on the surface of ferritin nanocages after reassembly. We need to develop more reliable drug payload methods to obtain defect‐free ferritin formulations. Second, the hydrophilicity and negative charge of the inner cavity of ferritin nanocages can lead to inefficient loading of negative and hydrophobic drugs. We need to study more efficient loading methods. Third, the half‐life of ferritin and its low tumor targeting to insufficient TfR1 expression require modification of ferritin to enhance its therapeutic effect. This could increase the complexity of industrial production and create new biosecurity concerns. At present, application of ferritin in tumor therapy is still in the preclinical stage. In the future, we should conduct clinical trials to further study the safety and efficacy of ferritin in patients. Although ferritin nanocarriers still have some problems as an emerging drug delivery system, the superior properties make it a promising strategy for cancer treatment.

## AUTHOR CONTRIBUTIONS

**Guodong Deng:** Writing – original draft (equal); writing – review and editing (equal). **Yang Li:** Writing – original draft (equal); writing – review and editing (equal). **Ning Liang:** Methodology (equal). **Pingping Hu:** Methodology (equal). **Yan Zhang:** Methodology (equal). **Lili Qiao:** Methodology (equal). **Yingying Zhang:** Methodology (equal). **Jian Xie:** Methodology (equal). **Hui Luo:** Methodology (equal). **Fei Wang:** Methodology (equal). **Fangjie Chen:** Methodology (equal). **Fengjun Liu:** Methodology (equal). **Deguo Xu:** Methodology (equal). **Jiandong Zhang:** Methodology (equal); supervision (equal); writing – review and editing (equal).

## FUNDING INFORMATION

This study was funded by the National Natural Science Foundation of China (no. 81803043) and Shandong Natural Science Foundation (ZR2021LSW023, ZR2021QH356 and ZR2022QH351).

## CONFLICT OF INTEREST STATEMENT

The authors declare that they have no conflict of interest.

## Data Availability

The data used in the study are available from the corresponding authors on reasonable request.
